# Sex and tissue specific gene expression patterns identified following de novo transcriptomic analysis of the Norway lobster, *Nephrops norvegicus*

**DOI:** 10.1186/s12864-017-3981-2

**Published:** 2017-08-16

**Authors:** Guiomar Rotllant, Tuan Viet Nguyen, Valerio Sbragaglia, Lifat Rahi, Kevin J. Dudley, David Hurwood, Tomer Ventura, Joan B. Company, Vincent Chand, Jacopo Aguzzi, Peter B. Mather

**Affiliations:** 1 Institut de Ciències del Mar (CSIC), Passeig Marítim de la Barceloneta, 37-49, 08003 Barcelona, Spain; 2 Faculty of Science, Health, Education and Engineering, GeneCology Research Centre, University of the Sunshine Coast, Sunshine Coast, QLD Australia; 3 Department of Biology and Ecology of Fishes, Leibniz-Institute of Freshwater Ecology and Inland Fisheries, Müggelseedamm, 310 Berlin, Germany; 4 Earth, Environmental and Biological Sciences, Science and Engineering Faculty, Queensland University of Technology, 2 George St, Brisbane, 4001 Australia

**Keywords:** Norway lobster, *Nephrops norvegicus*, Gene expression, Reproduction, RNA-Seq, Sex-specific expression, Sex-biased expression, Transcriptome

## Abstract

**Background:**

The Norway lobster, *Nephrops norvegicus*, is economically important in European fisheries and is a key organism in local marine ecosystems. Despite multi-faceted scientific interest in this species, our current knowledge of genetic resources in this species remains very limited. Here, we generated a reference de novo transcriptome for *N. norvegicus* from multiple tissues in both sexes. Bioinformatic analyses were conducted to detect transcripts that were expressed exclusively in either males or females. Patterns were validated via RT-PCR.

**Results:**

Sixteen *N. norvegicus* libraries were sequenced from immature and mature ovary, testis and *vas deferens* (including the masculinizing androgenic gland). In addition, eyestalk, brain, thoracic ganglia and hepatopancreas tissues were screened in males and both immature and mature females. RNA-Sequencing resulted in >600 million reads. De novo assembly that combined the current dataset with two previously published libraries from eyestalk tissue, yielded a reference transcriptome of 333,225 transcripts with an average size of 708 base pairs (bp), with an N50 of 1272 bp. Sex-specific transcripts were detected primarily in gonads followed by hepatopancreas, brain, thoracic ganglia, and eyestalk, respectively. Candidate transcripts that were expressed exclusively either in males or females were highlighted and the 10 most abundant ones were validated via RT-PCR. Among the most highly expressed genes were *Serine threonine protein kinase* in testis and *Vitellogenin* in female hepatopancreas. These results align closely with gene annotation results. Moreover, a differential expression heatmap showed that the majority of differentially expressed transcripts were identified in gonad and eyestalk tissues. Results indicate that sex-specific gene expression patterns in Norway lobster are controlled by differences in gene regulation pattern between males and females in somatic tissues.

**Conclusions:**

The current study presents the first multi-tissue reference transcriptome for the Norway lobster that can be applied to future biological, wild restocking and fisheries studies. Sex-specific markers were mainly expressed in males implying that males may experience stronger selection than females. It is apparent that differential expression is due to sex-specific gene regulatory pathways that are present in somatic tissues and not from effects of genes located on heterogametic sex chromosomes. The *N. norvegicus* data provide a foundation for future gene-based reproductive studies.

**Electronic supplementary material:**

The online version of this article (doi:10.1186/s12864-017-3981-2) contains supplementary material, which is available to authorized users.

## Background

As with many other commercially important aquatic species, a global decline has been reported for the Norway lobster, *Nephrops norvegicus* in recent years. According to the Food and Agriculture Organization (FAO), *N. norvegicus* captures declined from 75,999 t in 2007 to 54,762 t by 2014 [1]. A general decline in decapod crustacean fisheries around the world has been attributed to a diverse array of factors. Major drivers contributing to the steady decline in wild crustacean populations include: inadequate legislation for management of wild resources, high fishing quotas, decreases in individual sizes of the crustacean species targeted, and a general increase in worldwide demand for crustaceans [2]. In order to implement well-informed management plans for key harvested species, we need to develop a clear understanding of critical factors that influence wild population health and persistence. Factors range from understanding each species’ ecology through to individual physiological characteristics as well as understanding the molecular basis of the natural reproductive biology of each species. As an example, at the ecological level, understanding habitat preferences of key species can inform managers about the potential benefits of marine reserves, their minimum dimensions and boundaries. At the physiological level, understanding annual patterns of growth and reproduction can better inform fishing quota limits based on gender, size and season. At the molecular level, understanding natural reproductive cycles can assist development and implementation of wild restocking programs or potentially development of breeding programs that focus on single sex cohorts. As a recent example, farming of giant freshwater prawn (*Macrobrachium rosenbergii*) has been enhanced via molecular manipulation of sex so that either all-male or all-female populations can now be practiced. This development required molecular characterization of genetic sex markers [3], in addition to gene silencing of the Androgenic gland insulin-like hormone (IAG) in order to generate all-male populations [4], or introduction of androgenic gland cells to generate all-female populations [5]. Application of these molecular approaches to other farmed decapod crustacean species has yet to be validated.

Most decapod crustaceans studied to date are dioecious, sexually dimorphic and start to differentiate into their individual genetic sexes as larvae [6]. Current knowledge about the molecular pathways involved in regulating the processes of sex determination and sexual differentiation in decapod crustaceans has been reviewed only recently [7]. In brief, initiation of sexual development occurs via a sex-specific genetic cascade mediated via a chromosomal process of sex determination. Chromosomal mechanisms are not conserved and can include either homogametic (ZZ/ZW) or heterogametic (XY/XX) males. A number of sex determination-linked factors have been identified previously in some decapods including the master *Sex-determinant* in *Drosophila* (Sxl) and downstream mediators that include *Transformer* (TRA) 1 and TRA-2 as well as *Feminizer-1* (FEM-1). Identification of decapod copies of these genes were based on homology with sequences identified and characterized in the nematode *Caenorhabditis elegans* and arthropod model organisms. What is clear is that the sex determination pathway in decapods is far from being conserved and must have evolved independently numerous times. This makes it very difficult to trace master regulators in non-model organisms. In this sense, pursuit of evolutionarily potentially conserved key genes like *Double-sex* and *mab-3 related transcription factor* Dmrt could prove useful [8].

After genetic sex has been determined, a complex process of sexual differentiation follows, resulting in sex-specific phenotypic development. The effect of the male-specific androgenic gland (AG) is fundamental to male sexual differentiation. The AG is located close to the sperm duct or testis and is responsible for expression of insulin-like hormone that controls testicular development directly right through to emergence of male secondary-sexual characteristics. It also influences sexual behavior. The AG itself is regulated by the primary neuroendocrine center referred to as the X-organ-Sinus-gland complex (XO-SG) that is located in the eyestalk. XO-SG acts through the XO-SG – AG – Gonad axis and secretes a multitude of neurohormones including *gonad/vitellogenesis inhibiting hormone* (GIH/VIH) and *molt-inhibiting hormone* (MIH). GIH/VIH and MIH are believed to regulate IAG expression directly and have an ongoing inhibitory effect during sexual development and during reproduction. XO-SG secreted neurohormones also control sexual maturation that enable reproductive functionality in concert with additional regulatory factors (either peptides or neuromodulators) produced in the brain and thoracic ganglia (TG). Together they regulate secrete two lipid factors: i) the molt hormone ecdysone and ii) the metamorphosis-regulating hormone methyl farnesoate (MF) that are produced by the Y-organ and the mandibular organ, respectively [9]. The two factors are considered to have conserved key roles during arthropod gonadal maturation [10]. It is becoming clear however, that there are additional unknown regulatory genes and pathways (yet to be identified) involved in the process. Further investigation will be required therefore, to better understand the complete molecular basis for biological differences between male and female crustaceans in addition to resolving intricate sexual developmental pathways.

The Norway lobster constitutes one of the most important fisheries in European waters while being the most valuable harvested crustacean across the region. This species is closely monitored by the International Council for the Exploration of the Sea (ICES) under the Working Group on *Nephrops* Surveys http://www.ices.dk/community/groups/Pages/WGNEPS.aspx. Given perceived threats from over-exploitation and also its relative importance in regional fisheries, *N. norvegicus* biology, including reproduction, has been reviewed several times over recent decades [11–14]. In brief, *N. norvegicus* has a wide distribution in European waters, including both shallow and deep waters in the Atlantic Ocean and Mediterranean Sea. Despite an extensive natural distribution (or perhaps as a function thereof, i.e. there is no biogeographical barrier to inhibit inshore/offshore connectivity), no inter-populational genetic differentiation has been detected to date at any geographical scale examined. *N. norvegicus* is a decapod in the suborder Pleocyemata a group that is characterized by females carry eggs on their abdomen after fertilization from sperm deposited previously by a male during copulation and then stored in the female’s thelycum until spawning takes place. Females carry eggs on their abdomen for a period that varies between 6 (Mediterranean Sea) to 11 months (Iceland) after which embryos hatch and undergo larval development that involves three planktonic larval stages and a single benthic postlarval stage that lasts on average one month depending on water temperature. Reproduction as a consequence, takes place biannually (Iceland) or annually (Mediterranean Sea). Males and females are morphologically similar apart from their secondary sexual characteristics (i.e. claw allometry and presence of a petasma in males, and thelycum in females). Anatomy of the reproductive system in *N. norvegicus* has been described in detail for both males [15] and females [16].

Apart from a vast knowledge about the physiology of *N. norvegicus* reproduction, there is a major gap with respect to our understanding of the molecular mechanisms underlying regulation of reproduction. Recent advances in genomics technologies can help to fill this gap. The number of studies that have used next-generation sequencing technology (NGST) and microarrays on crustacean taxa has increased significantly in recent years. As examples, studies have been conducted recently on freshwater crayfish *Astacus astacus* [17], *Cherax cainii* [18], *Cherax destructor* [18], *Cherax quadricarinatus* [19–21], *Pontastacus leptodactylus* [22], *Procambarus clarkii* [23–26] and clawed lobsters *Homarus americanus* [27–30] and *N. norvegicus* [31], all members of the infraorder Astacidea. In this last study, two transcriptomes were published from eyestalk tissues from adult males maintained in a laboratory under dark or light conditions. Here, we generated a multi-tissue transcriptome from adult males, and mature and immature females to generate a reference library for the target species that will enhance a depauperate molecular toolkit for future studies directed at better resolving *N. norvegicus* physiology, development and reproduction.

Earlier studies of sex-biased transcriptomes of crustaceans have focused mainly on adult gonadal tissue, and reported several sex-specific transcripts including; an *Ovarian serine protease/nudel* homolog in the ovaries of the shrimp *Marsupenaeus japonicus* [32]; Female sterile and *Ovarian lipoprotein receptor* homologues in giant tiger shrimp *Penaeus monodon* [33], as well as *Dmrt* in the Chinese mitten crab *Eriocheir sinensis* [34]. A sex-biased gene expression analysis has also been conducted on whole individual samples of juvenile *E. sinensis* [35]. The authors classified 40 unigenes as testis or sperm-specific genes and 24 as ovary or oocyte-specific genes. Of the sex-biased genes identified in the juvenile transcriptomes, the study validated and showed that *Nit protein 2-like* (NIT2) was highly up-regulated in females and *CYP3A4* (Cytochrome P450, family 3, subfamily A, polypeptide 4) was up-regulated in males. Gao et al. [36] also validated 33 ovary-specific expressed transcripts and 14 testis-specific expressed transcripts, but the genes were not annotated.

The present study is the first relating to sex-specific transcripts in crustaceans that can lead to discovery of genomic-based sex markers with potential to shed new light on sex determination mechanisms. Here we screened five tissues (gonads, eyestalks, thoracic ganglia, brain and hepatopancreas) that target the most relevant genes involved with reproduction and in particular, sex-specific gene expression in *N. norvegicus*. The multi-tissue reference libraries from males, immature and mature females developed here will increase our knowledge about regulation of reproductive pathways and contribute greatly to planned future gene-based studies. Ultimately, these factors are likely to be of utmost importance when developing appropriate fisheries management tools that allow ongoing sustainable exploitation of wild *N. norvegicus* stocks.

## Methods

### Sampling and RNA extraction

Adult *N. norvegicus* were collected offshore from Barcelona harbour (Spain) from the trawling fishing vessel Maireta III. On board, male and female *N. norvegicus* were separated based on previously described external sexual characteristics [11]. Females were further separated into mature (stage IV) and immature (stage II) females, as previously described [16]. Tissues (testes, *vas deferens*, ovaries, hepatopancreas, muscle, eyestalks, brain and thoracic ganglia) were then dissected out and samples preserved in RNA-later (Ambion) and stored at −80 °C for later use. Samples were transported to the Molecular Genetics Research Facility at the Queensland University of Technology (Australia) for total RNA extraction, cDNA libraries synthesis, sequencing and downstream bioinformatics analyses., Samples of each tissue from 3 to 4 individuals were pooled to create a master pool. Total RNA was extracted using a modified TRIsure™ (Bioline, AU) protocol according to the manufacturer’s recommendations. Total RNA purity as well as concentration were checked using a spectrophotometer (Beckman Coulter) and Qubit RNA Assay Kit on the Qubit 2.0 Fluorometer (Life Technologies, USA). RNA integrity was evaluated using an Agilent Bioanalyzer 2100 (Agilent Technologies, CA, USA).

### Preparation of cDNA libraries and Illumina sequencing

Sequencing libraries were generated using Illumina TruSeq Stranded mRNA Sample Preparation Kit (Illumina Inc., CA, USA) following the manufacturer’s recommendations. In brief, poly-T-oligo magnetic beads supplied in the kit were used to capture mRNA from total RNA. Fragmentation was then carried out using divalent cations, followed by reverse transcription into first strand cDNA using reverse transcriptase and random primers. A second strand of cDNA was then synthesized using DNA polymerase I and RNAse. All products were further purified and amplified via PCR to generate the pre-sequencing cDNA libraries. Finally, all libraries preparations were sequenced on the Illumina NextSeq 500, resulting in the final 150 bp paired-end (PE) reads used in the study.

### Quality control and de novo assembly of the reference transcriptome

**Fig. 1:**
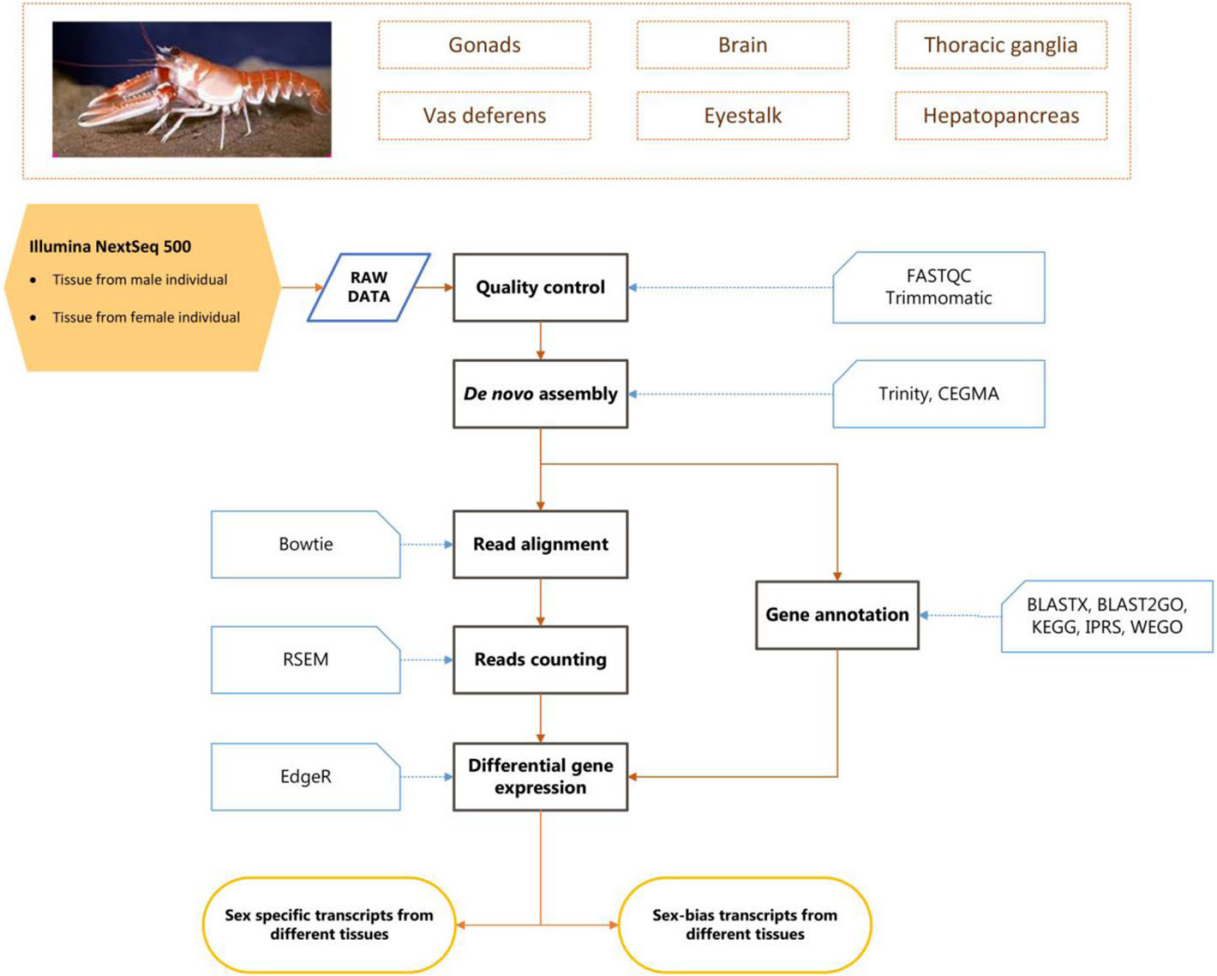
Bioinformatics workflow

Reads output from Illumina NextSeq in the form of FASTQ files were stored, backed up and assessed for quality using FastQC software [37]. Reads from the current dataset were merged with additional Illumina reads from a previous study (two eyestalk libraries adult male lobsters kept in captivity for six months were sampled at night and during the day) [31]. The bioinformatics workflow employed here is illustrated in Fig. 1. Quality assurance of reads was conducted using Trimmomatic [38]. The software was designed specifically for Illumina reads to identify and trim nucleotides falling below a pre-set threshold. The parameters used in the current study were LEADING = 5; TRAILING = 5; SLIDINGWINDOW = 3:10; MINLEN = 26; HEADCROP = 8. After trimming, all cDNA libraries were reassessed again for quality metrics (data not shown). All post quality control FASTQ files were then concatenated and de novo assembled using Trinity [39] on the Lyra HPC at Queensland University of Technology. Trinity was run on filtered PE reads applying a fixed k-mer value of 25, min_kmer_cov 2 and minimum contig length of 200 bp. The output FASTA file (the final reference transcriptome) was then tested for post-assembly metrics including N50 and total number and contig length. The final reference transcriptome was assessed for completeness using CEGMA (Core Eukaryotic Genes Mapping Approach) [40].

### Functional annotation of the reference transcriptome

All assembled contigs were scanned against the NCBI non-redundant database (nr) using BlastX algorithm with E-value threshold set at 1.00E-6. Blasted contigs were then loaded into BLAST2GO version 3.0 [41], an automated toolset used for characterizing large datasets for Gene Ontology (GO) annotation. Annotated transcripts were distributed into three ontology categories: molecular function, cellular component and biological process. A final annotation file was produced after GO-mapping, GO term assignment, annotation augmentation and a generic GO-Slim process. After obtaining GO annotation, Web Gene Ontology Annotation Plot (WEGO) software [42] was used to characterize the distribution of gene functions from the generated reference transcriptome.

### Mapping, reads counting and sex-specific transcripts evaluation

Trimmed reads from gonad, hepatopancreas, eyestalk, brain and thoracic ganglia dissected from wild caught mature males and mature females (not including the laboratory reared males and immature females) were mapped back to the reference transcriptome assembly using Bowtie applying default settings [43]. Read counts were generated using RSEM [44]. EdgeR software [45] was used to conduct differential gene expression analysis and to calculate false discovery rate of multiple-hypothesis testing. The following data filtering were used: *p*-value <0.001, FDR (false discovery rate) value <0.001 and at least 200 reads in either male or female. From the filtered data, a transcript to be considered as a sex-specific candidate had to have a read count of 0 in either male or female. To select for the most representative genes we chose the 30 most abundant transcripts from each sex and tissue.

### RT-PCR confirmation of sex-specific transcripts

Among abundantly-expressed sex-specific transcripts, we selected five per sex based on previous reports and GO annotation. We also selected a random transcript per sex from the 20 most abundant sex-specific transcripts. We tested the sex-specific transcripts using Reverse Transcriptase PCRs (RT-PCR) to amplify the cDNA product, followed by PCR using genomic DNA (gDNA) as a template.

#### RT-PCR

We used 3 samples from both males and females with each sample comprising a pool of tissue from 3 to 4 animals. RNA was extracted using the method described above. Selected tissues were gonads for all genes tested (*Serine threonine protein kinase,* STPK*; Venom c-type lectin mannose binding,* CTL*; Meprin,* MEP*; Slowpoke potassium channel family,* SLO*; Dual specificity mitogen-activated protein kinase,* MAPK*; Mannose-binding protein,* MBP*; Von Willebrand factor,* VWF; *Vitellogenin receptor*, VgR; *Meloxyperoxidase*, MPO) and hepatopancreas for *Vitellogenin*, Vg. As housekeeping (internal reference) genes, we used *Elongation factor 1* ***α*** (EF1-**α**) and *Glyceraldehyde 3-phosphate dehydrogenase* (GAPDH) since they have been validated previously in crustaceans [46, 47]. Specific primers for each gene were designed using Primer3 software (Additional file 1 **:** Table S1) [48]. In the case of genes with multiple transcripts, all transcripts were aligned to find the common region. Primers were then designed based on the common region to enable amplification of all potential isoforms. Prior to RT-PCR, residual DNA was removed from the RNA using the RQ1 RNase-Free DNase PROMEGA kit (Cat. # M6101). Then SensiFAST cDNA Synthesis Kit (BIOLINE; Cat. # BIO-65054) was used for reverse transcription, followed by PCR amplification with MyTaq HS DNA Polymerase (Cat. # BIO-21112). PCR settings were 95 °C for 1 min, followed by 30 cycles of 95 °C denaturation for 15 s, annealing temperature of 56 °C for 15 s and extension of 72 °C for 10s. Following PCR, products were loaded onto an agarose gel (1.5% agarose in Tris-Borate-EDTA buffer) with GelRed (Cat. # Biotium-41,003) and visualized under UV-light.

#### gDNA-PCR

Extraction of DNA from muscle tissue (3 samples from males and 3 samples from females - each sample represents muscle from a single animal) was conducted using the BIOLINE Isolate II genomic DNA kit (Cat. # BIO-52066). PCR settings were as described above for cDNA.

### PCR product sequencing

To validate amplification of each sex-specific gene, we sequenced PCR products to confirm that their sequence matched the sequence extracted from the transcriptomes. Amplicons were cleaned using Isolate II PCR and gel clean kits (BIOLINE; Cat. # BIO-52059) and then sequenced on an ABI 3500 Genetic Analyzer (Applied Biosystems, CA, USA). Geneious software v8.1 (Biomatters Ltd., New Zealand) was utilized for sequence analyses.

### Differential gene expression and sex-biased transcript evaluation

Previously performed mapping, read counting and differential gene expression (DGE) analyses (described above) served for sex-biased transcript analysis. The designated threshold for any transcript to be considered as significantly different between male and female was |log(Fold change)| > 2, *p*-value <0.001, FDR value <0.001. Heatmaps showing clusters of transcripts that were different between sexes for each tissue were illustrated using the analyse_diff_expr.pl scripts included in the Trinity de novo assembler [39]. Data were log2-transformed for the illustration and with an E-value threshold set at 1.00E-6. Two dimensional plots of principal components were calculated by performing principal component analysis (PCA) of the transposed log2-transformed FPKM values from male and female eyestalk, brain and thoracic ganglia samples using R software.

## Results

### Generation of a *Nephrops norvegicus* reference library

In total, our libraries resulted in more than 600 million reads from the Illumina NextSeq 500. That, combined with the previously generated Hiseq 2000 libraries, provided more than 800 million reads. On average, each library had more than 50 million reads with >90% of reads above the quality threshold for all libraries. All raw reads and quality control statistics are presented in Table 1.

**Table 1:** Raw reads and quality control of reads for *N. norvegicus* libraries

Tissue	Sex	Number of raw reads	Raw read length	%GC	Number of reads after trimming	Read length after trimming
Testis	M	47,454,625	151	44	44,384,279	26–143
Ovary	MF	56,859,898	151	43	53,448,056	26–143
	IF	52,854,378	151	48	49,655,513	26–143
Vas Deferens	M	49,222,546	151	43	46,802,691	26–143
Hepatopancreas	M	56,240,204	151	43	52,803,977	26–143
	MF	54,715,141	151	46	50,574,033	26–143
	IF	52,217,561	151	46	48,752,848	26–143
Eyestalk	M	44,910,345	151	43	41,968,679	26–143
	MF	45,799,203	151	42	42,121,851	26–143
	IF	53,668,850	151	42	49,410,048	26–143
Brain	M	58,405,643	151	42	54,423,042	26–143
	MF	67,530,741	151	42	62,997,606	26–143
	IF	61,357,339	151	42	57,343,088	26–143
Thoracic ganglia	M	47,227,119	151	41	43,723,658	26–143
	MF	58,418,219	151	43	54,460,212	26–143
	IF	49,303,270	151	42	45,710,029	26–143
Data from a previous study [7]						
Eyestalk	ML	87,830,082	101	41	84,425,620	26–89
	MD	91,938,198	101	44	87,970,542	26–89

*IF* immature female; *M* Male; *MD* Male maintained in Darkness; *MF* mature female; *ML* Male maintained in Light conditions

De novo transcriptome assembly of the current dataset using the Trinity assembler generated 333,225 transcripts, with an average transcript size of 708 base pairs (bp) and N50 of 1272 bp. Total size of the reference transcriptome was 235,992,830 bp. All transcripts were longer than 200 bp and we recorded more than 51,000 transcripts longer than 1000 bp. To assess the completeness of transcriptome assembly, we employed CEGMA packages resulting in 97.53% completeness, indicating the high quality de novo assembly. A brief summary of de novo assembly statistics are provided in Table 2.

**Table 2:** De novo assembly statistics of the *N. norvegicus* reference transcriptome

Number of transcripts	333,225
Total size of transcripts (nt)	235,992,830
Longest transcripts (bp)	33,925
Number of transcripts >1 k bp	51,881
Number of transcripts >10 k bp	394
Mean transcript size (bp)	708
N50 transcript length (bp)	1272
Transcriptome Assembly Completeness (%)	97.53

Gene identification was conducted after generation of the reference transcriptome, BLAST searches were conducted using the BlastX algorithm against the Nr (non-redundant protein sequences in NCBI). Only 37,296 transcripts (11%) showed significant hits against known proteins. Of these, only 20,595 (6%) transcripts could be fully annotated with GO available data, illustrating the scarcity of crustacean sequences in current genomic databases. The most abundant BLAST hits were associated with a widely studied crustacean species *Daphnia pulex* (9%), and then several insect species *Tribolium castaneum* (6%), *Pediculus humanus* (4%), *Strongylocentrotus purpuratus* (3%) and *Acyrthosiphon pisum* (3%). The highest hits for species distribution were associated with unknown species (48%) (Additional file 1: Table S1).

**Fig. 2:**
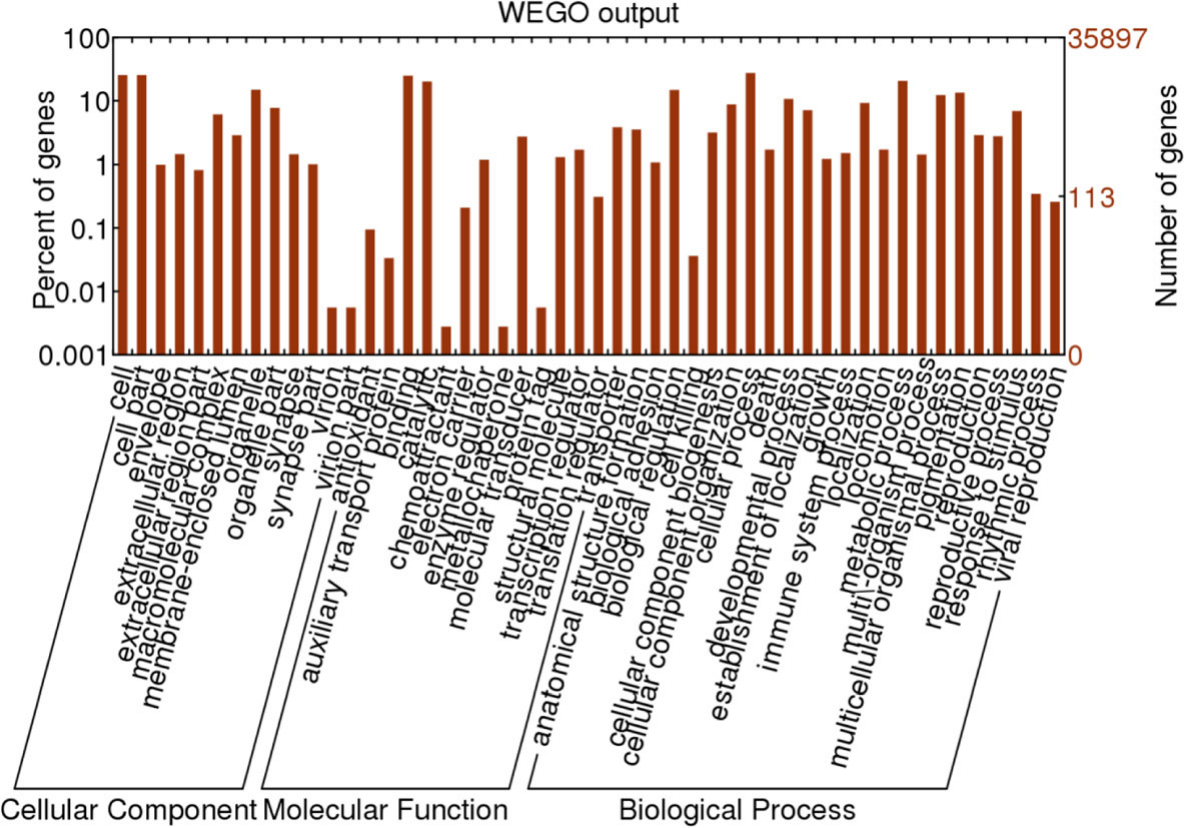
Distribution of GO terms

We also generated a GO annotation plot to visualize the distribution of GO terms in our reference transcriptome. The percentages of annotated *N. norvegicus* sequences assigned to GO terms are shown in Fig. 2. Analysis of GO term distribution showed that cell (GO:0005623), binding (GO:0005488) and cellular process (GO:0009987) were the most common annotation terms within the three GO categories.

### Sex-specific transcripts analyses

**Fig. 3:**
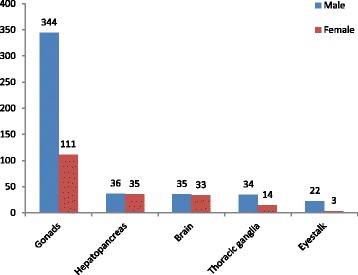
Comparative sex-specific transcripts among different tissues

In the current study, we detected a large number of transcripts specifically expressed in male but not in female tissues (and vice-versa; Additional file 1: Table S1). In total, the number of sex-specific transcripts (male-specific numbers / female-specific) were 344/111 in the gonads, 36/35 in the hepatopancreas, 35/33 in the brain, 34/14 in the thoracic ganglia, and finally 22/3 in the eyestalk. Sex-specific transcripts in each tissue are presented in Fig. 3 showing that males always had a higher number of transcripts expressed exclusively compared with females.

The most abundant gonad-specific transcripts included *Serine threonine protein kinase, Venom c-type lectin mannose binding protein, Dual specificity mitogen-activated protein kinase, Meprin protein* and *Slowpoke potassium channel protein* in males; while in females the most abundant gonad-specific transcripts were *Mannose-binding protein, Von Willebrand factor, Myeloperoxidase* and *Vitellogenin* receptor. In the hepatopancreas, the main female-specific transcript found was *Vitellogenin* with eight different isoforms of Vg identified. Parameters for the entire list of sex-specific transcripts are listed in supplementary material (Additional file 2: Table S2).

**Fig. 4:**
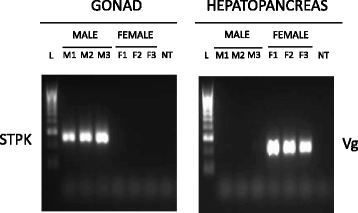
Expression patterns of the highest sex-specific genes

The ten most highly differentially expressed transcripts were subjected to additional analyses. Transcript information, including GO classification, is detailed in Table 3. All sex-specific transcripts tested were found to be specific to a single tissue as well. Validation of expression sex-specific transcripts (cDNA) as well as gDNA amplification are presented in Table 4. Expression profiles of the highest expressed validated sex-specific transcripts in *N. norvegicus* (STPK in the male gonads and Vg in the female hepatopancreas) are illustrated in Fig. 4. cDNA expression of CTL, MEP, SLO and MAPK were validated in testes while VgR, VWF, MBP and MPO were validated as being specific to ovaries (Additional file 3: Fig. S2). House keeping genes (EF1α and GAPDH) expression was validated in both gonad and hepatopancreas tissues (Additional file 3: Fig. S2). Sanger sequencing of gDNA in muscle tissue confirmed the presence of all sex-specific gene sequences apart from VWF. Failure to amplify VWF may have resulted from presence of associated introns.

**Table 3:** Highly expressed sex-specific transcripts in *N. norvegicus*

Sex-specific	Gene	Tissue	Contig #	# reads mapped	FPKM	*P*-value	GO annotation
Male	STPK	Testes	c207207_g2_i1	123,796	7963	1.68E-37	*F*: ATP binding; *F*: protein kinase activity; *P*: phosphorylation; *F*: protein serine/threonine kinase activity; *P*: protein phosphorylation; *F*: kinase activity
	CTL	Testes	c121960_g1_i1	20,001	1338	1.37E-29	*F*: carbohydrate binding; *P*: biological_process; *C*: cellular_component
	MEP	Testes	c16959_g1_i2	15,261	1108	2.04E-28	*F*: hydrolase activity
	SLO	Testes	c158376_g1_i1	13,219	402	8.56E-28	*F*: calcium-activated potassium channel activity; *F*: voltage-gated potassium channel activity; *C*: membrane; *F*: large conductance calcium-activated potassium channel activity; *P*: potassium ion transport; *C*: voltage-gated potassium channel complex; *F*: metal ion binding; *P*: potassium ion transmembrane transport; *F*: protein binding
	MAPK	Testes	c206116_g1_i1	15,261	1050	1.73E-29	*F*: transferase activity, transferring phosphorus-containing groups
Female	Vg	Hepatopancreas	c208407_g1_i1	333,838	2843	1.36E-44	*P*: lipid transport; *F*: lipid transporter activity; *P*: oogenesis
	MBP	Ovary	c208884_g1_i2	5719	1075	6.26E-30	*F*: carbohydrate binding
	VWF	Ovary	c207785_g1_i1	6210	191	4.03E-28	*F*: calcium ion binding; *F*: metal ion binding; *P*: oxidation-reduction process; *F*: oxidoreductase activity; *P*: metabolic process; *F*: chitin binding; *P*:chitin metabolic process; *P*: cell adhesion; *C*: extracellular region
	VgR	Ovary	c208891_g2_i1	2856	64	9.36E-25	*F*: calcium ion binding; C:integral component of membrane; *C*: membrane; *F*: receptor activity; *P*: receptor-mediated endocytosis; *P*: endocytosis; *C*: cellular_component
	MPO	Ovary	c179405_g1_i1	3761	136	6.01E-26	*P*: oxidation-reduction process; *F*: heme binding; *P*: response to oxidative stress; *F*: peroxidase activity

*CTL* Venom c-type lectin mannose binding; *MAPK* Dual specificity mitogen-activated protein kinase; *MBP* Mannose-binding protein; *MEP* Meprin; *MPO* Meloxyperoxidase; *SLO* Slowpoke potassium channel family; *STPK* Serine threonine protein kinase; *Vg* Vitellogenin; *VgR* Vitellogenin receptor; *VWF* Von Willebrand factor

**Table 4:** Expression profile of validated sex genes in *N. norvegicus*

	mRNA (gene expression)		gDNA (amplification)	
GENES	Male	Female	Male	Female
Serine threonine protein kinase	+	−	+	+
Venom c-type lectin mannose binding	+	−	+	+
Meprin	+	−	+	+
Slowpoke potassium channel family	+	−	+	+
Mitogen-activated protein kinase	+	−	+	+
Vitellogenin	−	+	+	+
Mannose-binding protein	−	+	+	+
Von Willebrand factor	−	+	−	−
Vitellogenin receptor	−	+	+	+
Meloxyperoxidase	−	+	+	+
Elongation factor 1 α	+	+	*	*
Glyceraldehyde 3-phosphate dehydrogenase	+	+	*	*

+: amplified successfully; −: not amplified; *: not applicable

### Sex-biased gene differential expression analyses

While the primary aim of the study was to analyze transcripts that were specific to either males or females, a general evaluation of all sex-biased transcripts was generated, providing an overview of the entire suite of differentially expressed transcripts between sexes (including the sex-specific transcripts, Additional file 4: Table S3).

**Fig. 5:**
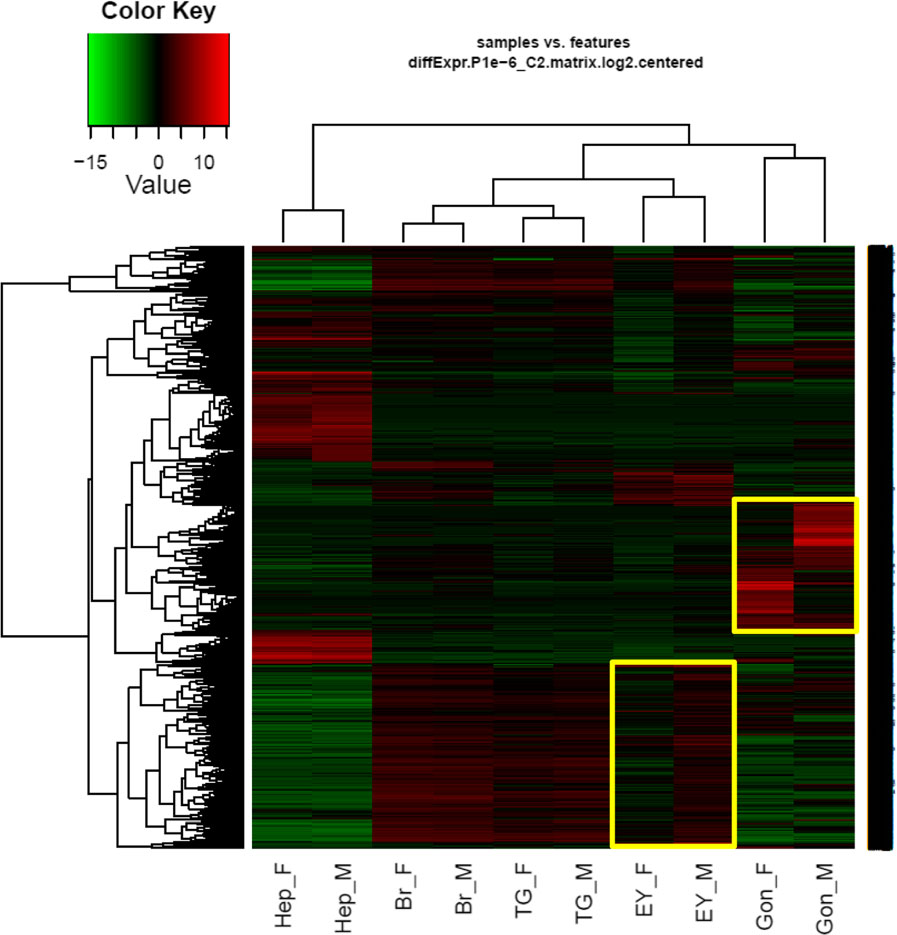
Heatmap of differentially expressed genes between sexes. Yellow boxes represents cluster of genes that are highly differentiated between males and females

A heatmap illustrating differential expression patterns of transcripts from multiple tissues (Fig. 5) highlights clusters of transcripts with significant differential expression patterns between males and females. As expected, most differentially-expressed transcripts were detected in the gonads of both sexes. When considering the differentially expressed genes presented in isolation (Fig. 5), the male eyestalk expression profile was similar to brain and thoracic ganglia in both males and females, while female eyestalk showed a very different expression profile compared with the remaining nervous system libraries, a result that was also evident in the PCA analysis (Additional file 5: Fig. S3). Hepatopancreas did not show a large number of transcripts differentially expressed between the sexes.

## Discussion

A *N. norvegicus* transcriptome was generated to investigate the molecular basis for reproduction in this species but also provides a platform for more general functional genomic studies of Norway lobster. Applications for the transcriptomic data can inform future efforts directed at restocking programs for *N. norvegicus* to support a declining fishery. From an ecological perspective, this transcriptome can also be a valuable resource for assessing differential gene expression patterns as a consequence of natural variation in habitat conditions because *N. norvegicus* possesses a very broad distribution in European waters both in shallow and deep-waters where populations are not structured geographically. The multi-tissue transcriptomic (18 transcriptomes merged from 6 tissues from males and 5 tissues from females) reference library generated here presents a high quality database (N50 of 1272 bp, average contig length of 708 bp and 97.53% of CEGMA) allowing deep mining for key factors associated with regulation of reproduction. Assembly numbers are comparable with recent attempts to generate reference transcriptomes for Astacid species [17, 18, 22, 24, 29]. With regard to CEGMA, our results show a slightly higher percentage compared with those for a recently published study of a freshwater decapod, *C. quadricarinatus* (94.76%) but average contig length in our study was shorter [21].

We identified 471 male-specific and 196 female-specific expressed transcripts in the *N. norvegicus* transcriptome with gonads in both sexes expressing the highest numbers of sex-specific transcripts compared with other sampled tissues. A similar outcome was observed for *M. japonicus* [32]. In addition, the higher number of testis versus ovary up-regulated transcripts found in *N. norvegicus* was a similar result to that obtained for *E. chinensis* [36], and the Eastern spiny lobster *Sagmariasus verreauxi* [49]. This implies that female gonadal development may require a smaller number of up-regulated transcripts compared with males. This could be due to the effect of male-specific IAG which is known to drive masculinization in crustaceans [7]. If the bias in majority male-specific expression observed for *N. norvegicus* is confirmed, this may result from adaptive changes in males, implying that males may in general, experience stronger selection than females.

Of the 667 transcripts identified in the Norway lobster, 445 were either male or female-specific. From these, we selected ten annotated to sex-related pathways for further validation. Serine threonine protein kinases were found to be highly expressed in the testes of *E. sinensis* [50]; the authors suggesting that STPKs may play a key role in both mitotic and meiotic divisions and extensive cellular re-modelling through cell cycle phases during spermatogenesis. In *M. rosenbergii*, a male reproductive-related protein homologous with STPK was found to be expressed exclusively in the sperm ducts as a protein with a putative role in phosphorylation and with a function involved with sperm capacitation and/or fertilization [51]. In *N. norvegicus*, 24 transcripts annotated as STPK were detected exclusively in the testes suggesting that several isoforms could have different functions in male reproduction (Additional file 4: Table S3).

The Mitogen-activated protein kinase cascade is also involved in numerous male reproductive processes in mammals, including spermatogenesis, sperm maturation and activation, capacitation and acrosome reaction prior to fertilization of the oocyte [52]. Recently, cDNAs of c-Jun N-terminal MAPKs in *E. sinensis* showed relatively high expression in the testis and their expression were found to decrease gradually over time during spermatogenesis [53]. MAPK- extracellular signal-regulated kinase 2 element has also been identified in the green mud crab (*Scylla paramamosain*) and the giant tiger shrimp (*P. monodon*), but in contrast in these species it has been implicated as playing a crucial role in ovary development [54, 55]. Based on the transcription pattern specifically expressed in *N. norvegicus* testis, we suspect that the MAPK transcript in the present study, may be related to the c-Jun N-terminal kinases subfamily as reported for *E. sinensis*. In earlier studies, Laufer and Biggers [56] suggested a possible role for insect juvenile hormone and/or methyl farnesoate in activation of MAPK during reproduction and morphogenesis. Both studies implicate involvement of MAPK pathways during crustacean reproduction.

C-type lectins are a family of calcium-dependent carbohydrate-binding proteins that are believed to play important roles in innate immunity in crustaceans [57, 58]. In mammalian reproductive physiology, CTLs are involved in molecular mechanisms that underlie successful fertilization of embryos, a process via which sperm lectins recognize specific carbohydrates present on glycoproteins on the egg surface [59]. C-type lectins that mediate sperm-egg recognition and fusion during fertilization have also been detected in sperm and soluble extracts from adult males, but not females in the hookworm *Ancylostoma ceylanicum* [60]. Meprins are unique proteases in the astacin family of metalloendopeptidases, and indeed more widely across the animal kingdom. They have an oligomeric structure and are disulfide-linked dimers that are highly glycosylated. Meprins have many attributes of receptors or integrins with adhesion, epidermal growth factor-like and transmembrane domains [61]. Meprins represent excellent models of hetero- and homo-oligomeric enzymes that are regulated at the transcriptional and posttranslational levels. The crayfish enzyme, Astacin, was the first Meprin characterized and is one of the smallest members of the family [62]. Astacin metalloprotease has been identified in male ticks *Dermacentor variabilis* as enzymes important in seminal fluid and are considered to be necessary for processing of male accessory gland proteins following insemination of the female [63]. Following evidence from insects, we suggest that CTLs may potentially play a role in sperm-egg recognition in *N. norvegicus* while Meprins could be related to mating activity.

The *Slowpoke* locus encodes a calcium-activated potassium channel in animals. In *Drosophila*, this ion channel can mediate courtship behavior (males vibrate their wings to produce a ‘song’ that attracts females), influencing individual reproductive success [64]. In mammals, SLO is localized in the sperm flagellum and during fertilization this channel increases sperm intracellular calcium levels and membrane hyperpolarization [65]. We found SLO in *N. norvegicus* to be expressed exclusively in the testis. To our knowledge, this is the first record of exclusive expression of SLO in a single sex in a decapod crustacean. SLO has only recently been identified as a differentially expressed gene in both abdominal and cardiac nervous system tissues but not in gonads (gonads were not tested in the cited study) in American lobster *H. americanus* [29].

As for transcripts exclusively expressed in female *N. norvegicus*, RT-PCR results showed that *Vitellogenin* mRNA can be detected only in the hepatopancreas of females. This pattern is similar to that found in the freshwater edible crab *Oziothelphusa senex* where Vg can also only be found in the hepatopancreas but not in other tissues including eyestalk, Y-organ, mandibular organ, thoracic ganglion, hypodermis or ovary [66]. This suggests that Vg expression is sex-specific during secondary vitellogenesis when large amounts of yolk are required over a short period of time to provide exogenous reserves in mature oocytes. *Vitellogenin receptor* and *Von Willebrand factor* are also related to Vg. *Vitellogenin* is taken into developing oocytes from the hemolymph by VgR via receptor-mediated endocytosis [67]. *Vitellogenin receptor* was expressed in the ovary but not in testis in both *P. monodon* juveniles and adult and expression levels in ovaries were significantly higher in adults compared with juveniles. This gene is also up-regulated during the final stage of ovarian development in adults [68]. Expression levels of genes with roles in ovarian development were not different between wild and domesticated *P. monodon* females, suggesting that Vg and VgR expression level is not related to broodstock condition and both influence maturation [69]. The D domain of mammalian VWF contains a similar sequence to that present in a freshwater crayfish *Pacifastacus leniusculus*. VWF is a clotting protein in the hepatopancreas and its sequence is homologous with Vg [70]. During maturation, oocytes consist mainly of yolk of which, the primary components are lipids. Lipid and lipoprotein oxidation have been documented using model systems containing purified myeloperoxidase in humans [71]. Presence of *Myeloperoxidase* in *H. americanus* hemocytes was reported by Anderson and Beaven [72]. *Myeloperoxidase* is a heme-containing protein present in the hemocyte that converts hydrogen peroxide into hypochlorous acid. This reaction could be related to the ability of activated cells to produce antimicrobial reactive oxygen species.

In all Pleocyemate decapods, including *N. norvegicus*, the female just after laying her eggs, kneads them with her ovigerous setae. Pleocyemate decapods are known to contain high levels of N-glycosylation sites on the proteoglycans associated with egg brooding. In blue swimmer crab *Portunus pelagicus*, *Mannose-binding protein* has been implicated in glycosylation [73]. Importance of glycosylation by MBP lies in a putative role in folding, processing and transport of this protein to the egg yolk as has been observed in *M. rosenbergii* [74].

A number of earlier studies have highlighted certain sex-specific transcripts including *Ovarian serine protease/nudel* homolog [32], *Female sterile* and *Ovarian lipoprotein receptor* [33], *Double-sex mab-3 related transcription factor* [34] and Nit protein 2-like and CYP3A4 [35]. In *N. norvegicus*, these genes were not identified as sex exclusive transcripts applying the designated threshold employed here. We did however, identify ovary-biased expression of *ovarian serine protease/nudel* homolog, *female sterile* and *lipoprotein receptor*. In addition, one DM-domain containing protein was predominantly expressed in the testis (Additional file 6: Table S4). It is also important to note that a relatively large number of sex-specific as well as sex-biased transcripts were detected in *N. norvegicus* and can be found in supplementary material (Additional file 3: Table S2 and Additional file 4: Table S3). Clustering of relative gene expression patterns using the heatmap approach illustrated that gonads and eyestalks were the tissues that showed the largest differences in sex-biased transcripts in *N. norvegicus *(Additional file 7: Figure S1). This is true despite the fact that *Vitellogenin* in the hepatopancreas was the most highly expressed transcript in females, but apart from Vg, this tissue did not show a large number of transcripts differentially expressed between the sexes. Future investigations would therefore benefit from focusing mainly on transcripts differentially expressed in the gonad and eyestalk between sexes. These results also indicate that sex-specific gene regulatory pathways exist in somatic tissues in Norway lobster. Furthermore, observation that all validated genes were present in the genome of both sexes suggest that differences in gene expression between the sexes result from differences in gene regulation and not from effects of genes located on heterogametic sex chromosomes. This approach could serve as a template for future gene based studies related to sex differences in *N. norvegicus*.

## Conclusions

- A reference transcriptome library was developed for *N. norvegicus* that has applications in future biological, wild restocking and fisheries studies.
- Sex-specific markers were mainly expressed in males in the 5 tissues tested implying that males may in general, experience stronger selection than females.
- Five sex-specific potential markers were identified in adult males (*Serine threonine protein kinase, Venom c-type lectin, Mannose binding, Meprin* and *Slowpoke potassium channel protein*) and females (*Vitellogenin, Mannose-binding protein, Von Willebrand factor, Vitellogenin receptor* and *Meloxyperoxidase*). All transcripts were sex-specific in the gonads except for *Vitellogenin* that was most highly expressed in the hepatopancreas.
- Differential expression among tissues revealed that tissues that possessed the largest numbers of sex-biased transcripts were the gonads and eyestalk with eyestalk showing higher expression levels in females.
- Sex-specific gene regulatory pathways exist in somatic tissues and not from effects of genes located on heterogametic sex chromosomes in the Norway lobster.

## Additional files

Additional file 1: Table S1. RT-PCR primers for sex-specific transcripts and housekeeping genes. CTL: Venom c-type lectin mannose binding; EF 1-a: Elongation factor 1- a; GAPDH: Glyceraldehyde 3-phosphate dehydrogenase; MBP: Mannose-binding protein; MEP: Meprin; MAPK: Dual specificity mitogen-activated protein kinase; MPO: Meloxyperoxidase; SLO: Slowpoke potassium channel family; STPK: Serine threonine protein kinase; Vg: Vitellogenin, VgR: Vitellogenin receptor; VWF: Von Willebrand factor. (DOCX 15 kb)

Additional file 2: Table S2. Full list of sex-specific transcripts. (XLSX 1280 kb)

Additional file 3: Figure S2. Gel results of validated sex-specific transcripts. (PDF 506 kb)

Additional file 4: Table S3. Full list of differentially expressed transcripts between sexes. (XLSX 853 kb)

Additional file 5: Figure S3. PCA of sex-biased genes. (PDF 8 kb)

Additional file 6: Table S4. List of differentially expressed transcripts between sexes reported in other studies identified in *N. norvegicus*. (XLSX 11 kb)

Additional file 7: Figure S1. Species distribution of blast hits. (DOCX 93 kb)

## Abbreviations

AG: Androgenic gland
Bp: Base pairs
CEGMA: Core Eukaryotic Genes Mapping Approach
CTL: Venom c-type lectin mannose binding
CYP3A4: Cytochrome P450, family 3, subfamily A, polypeptide 4
DGE: Differential gene expression
Dmrt: Double-sex and mab-3 related transcription factor
EF1-α: Elongation factor 1 α
FAO: Food and Agriculture Organization
FDR: false discovery rate
FEM-1: Feminizer-1
GAPDH: Glyceraldehyde 3-phosphate dehydrogenase
GIH/VIH: gonad/vitellogenesis inhibiting hormone
GO: Gene Ontology
IAG: Androgenic gland insulin-like hormone
ICES: International Council for the Exploration of the Sea
MAPK: Dual specificity mitogen-activated protein kinase
MBP: Mannose-binding protein
MEP: Meprin
MF: methyl farnesoate
MIH: molt-inhibiting hormone
MPO: Meloxyperoxidase
NCBI: National Center for Biotechnology Information
NGST: next-generation sequencing technology
NIT2: Nit protein 2-like
nr: Non-redundant
PE: Paired-end
PMTST1: *P. monodon* testis-specific transcript 1
RT: Reverse Transcriptase
SLO: Slowpoke potassium channel family
STPK: Serine threonine protein kinase
Sxl: Sex-determinant
TG: Thoracic ganglia
TRA 1: Transformer 1
TRA-2: Transformer 2
Vg: Vitellogenin
VgR: Vitellogenin receptor
VWF: Von Willebrand factor
WEGO: Web Gene Ontology
XO-SG: X-organ-Sinus-gland complex
