# Supplementary material for: Sex and tissue specific gene expression patterns identified following de novo transcriptomic analysis of the Norway lobster, Nephrops norvegicus
Source: BMC Genomics. 2017 Aug 16;18:622. doi: 10.1186/s12864-017-3981-2 (PMC5559819; doi:10.1186/s12864-017-3981-2)
Supplement: Supplementary file 1 — RT-PCR primers for sex-specific transcripts and housekeeping genes. CTL: Venom c-type lectin mannose binding; EF 1-a: Elongation factor 1- a; GAPDH: Glyceraldehyde 3-phosphate dehydrogenase; MBP: Mannose-binding protein; MEP: Meprin; MAPK: Dual specificity mitogen-activated protein kinase; MPO: Meloxyperoxidase; SLO: Slowpoke potassium channel family; STPK: Serine threonine protein kinase; Vg: Vitellogenin, VgR: Vitellogenin receptor; VWF: Von Willebrand factor. (DOCX 15 kb) [file 12864_2017_3981_MOESM1_ESM.docx]

Table S1 RT PCR primer of the sex-specific genes of *N. norvegicus* and housekeeping genes.

| **Gene** | **Forward primer** | **Reverse primer** |
| --- | --- | --- |
| STPK | TGCTGACGCACAAGGTAGG | AACAGGGCTCTTTGGATGAA |
| CTL | GTCTGCTGTGATGGGTGCT | CGTTGTGGTGGAGTTCTTTG |
| MEP | AGTTGCTCTTGGTCGCTTTG | CCTCGTCTTCTGGGTCGTT |
| SLO | ACACGGCCTGGATAGCAACAT | AGAGCAGTATTCCCAAGCATT |
| MAPK | CATTCCTTGACCGCCTACAC | GTCTCTGACTCCGTCCACAA |
| Vg | TGGCAGGATGAACAGCAATAT | TGCTGATGAACGGACTCAAAA |
| VgR | AAATGGTCACACACGCTCAC | CGGTCGCTGTAATGAAACCT |
| VWF | GCGGAAGAGCCAGAATACAA | ACACCACCACCCCAACAC |
| MBP | CGTCTGCGTTGTCTGGTTG | TTCGTGGCTGGAGTTCTTTT |
| MPO | AAGAAGGCAGGGTTGAGTTG | GCGTGAAAAGTGGCGTAGA |
| EF1-α | CAACAAGATGGACAGCACAGA | CAACAAGATGGACAGCACAGA |
| GAPDH | AGTCCCCTCGCAACACCT | CCATCCTCCATCTTCACCTC |

CTL: Venom c-type lectin mannose binding; EF1-α: Elongation factor 1- α; GAPDH: Glyceraldehyde 3-phosphate dehydrogenase; MBP: Mannose-binding protein; MEP: Meprin; MAPK: Dual specificity mitogen-activated protein kinase; MPO: Meloxyperoxidase; SLO: Slowpoke potassium channel family; STPK: Serine threonine protein kinase; Vg: Vitellogenin, VgR: Vitellogenin receptor; VWF: Von Willebrand factor.
