# Supplementary figures and images for: Sex and tissue specific gene expression patterns identified following de novo transcriptomic analysis of the Norway lobster, Nephrops norvegicus
Source: BMC Genomics. 2017 Aug 16;18:622. doi: 10.1186/s12864-017-3981-2 (PMC5559819; doi:10.1186/s12864-017-3981-2)

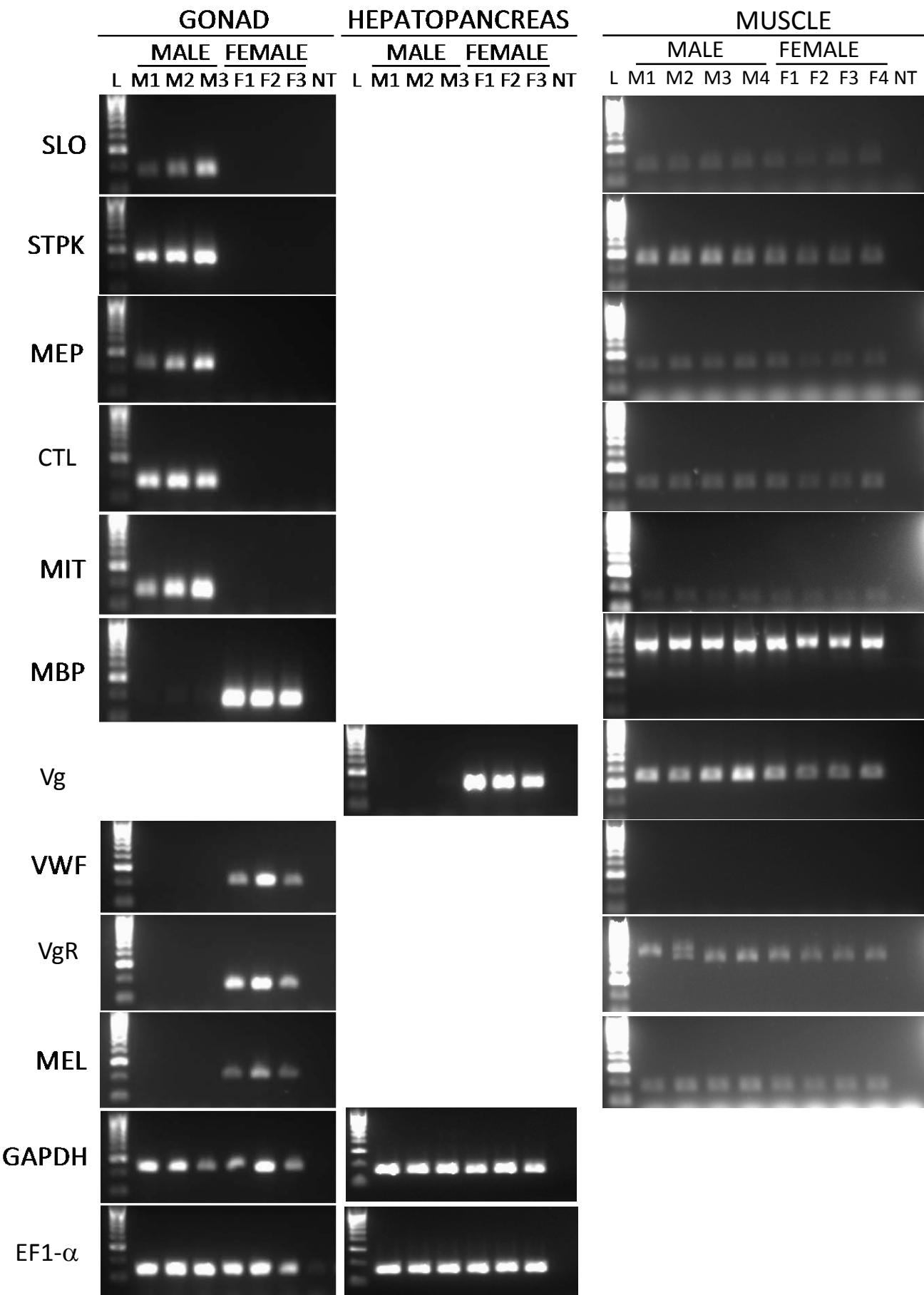

Supplement: Supplementary file 3 — Gel results of validated sex-specific transcripts. (PDF 506 kb) [file 12864_2017_3981_MOESM3_ESM.pdf]

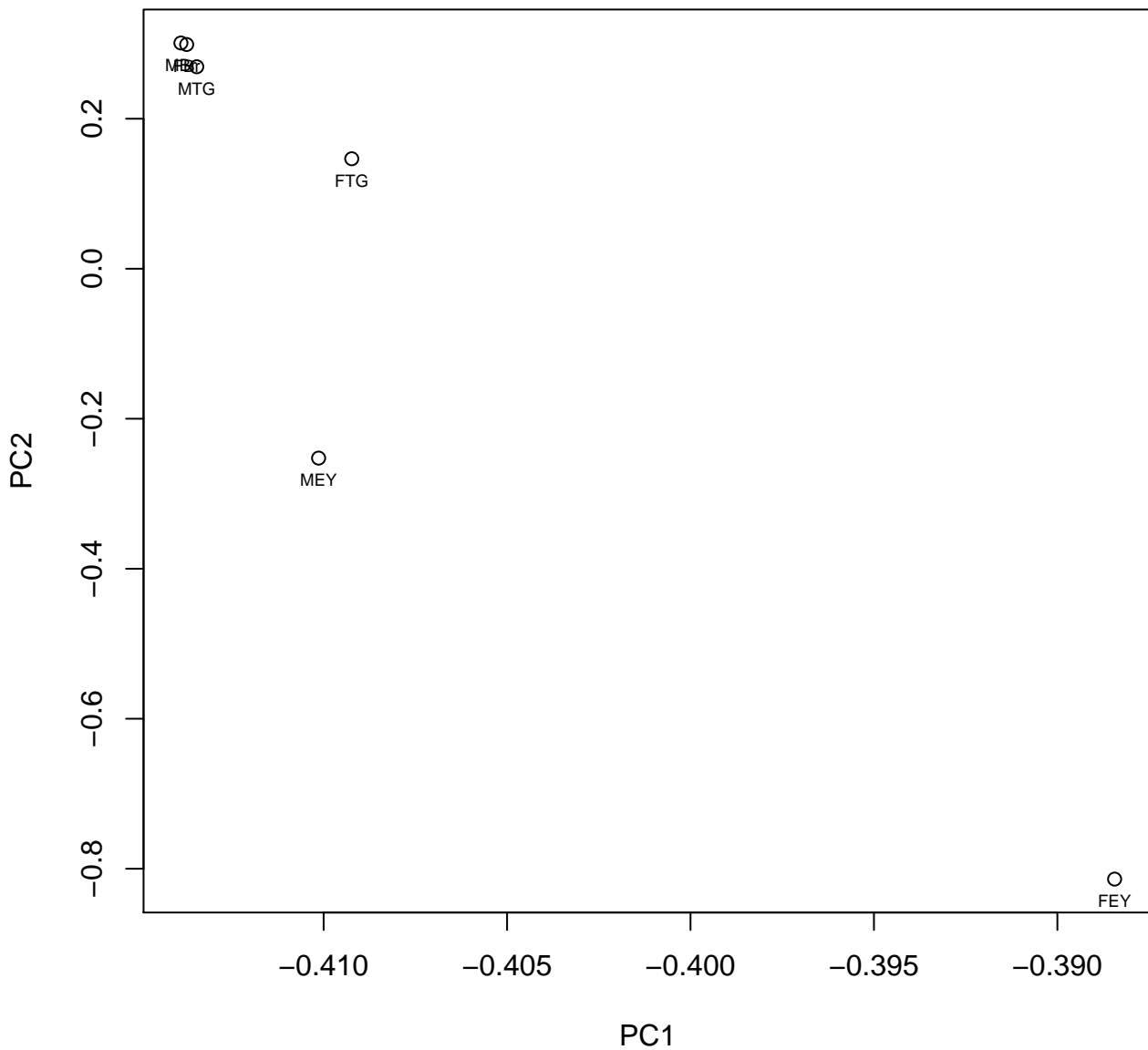

Supplement: Supplementary file 5 — PCA of sex-biased genes. (PDF 8 kb) [file 12864_2017_3981_MOESM5_ESM.pdf]
