# Supplementary material for: Sex and tissue specific gene expression patterns identified following de novo transcriptomic analysis of the Norway lobster, Nephrops norvegicus
Source: BMC Genomics. 2017 Aug 16;18:622. doi: 10.1186/s12864-017-3981-2 (PMC5559819; doi:10.1186/s12864-017-3981-2)
Supplement: Supplementary file 7 — Species distribution of blast hits. (DOCX 93 kb) [file 12864_2017_3981_MOESM7_ESM.docx]

Fig. 2 Species distribution of blast hits from *N. norvegicus* reference library
